# A nomogram for predicting neonatal acute respiratory distress syndrome in patients with neonatal pneumonia after 34 weeks of gestation

**DOI:** 10.3389/fped.2024.1451466

**Published:** 2025-01-09

**Authors:** Aosong Yu, Huanhuan Hou, Lingyi Ran, Xiaojia Sun, Wanchun Xin, Tong Feng

**Affiliations:** ^1^ Department of Pediatrics, Dandong Central Hospital, China Medical University, Dandong, China; ^2^ School of Clinical Medicine, Xinjiang Medical University, Urumqi, China

**Keywords:** neonatal acute respiratory distress syndrome, late-preterm infants, full-term infants, prediction nomogram, neonatal pneumonia

## Abstract

**Objective:**

To establish a prediction nomogram for early prediction of neonatal acute respiratory distress syndrome (NARDS).

**Methods:**

This is a retrospective cross-sectional study conducted between January 2021 and December 2023. Clinical characteristics and laboratory results of cases with neonatal pneumonia were compared in terms of presence of NARDS diagnosis based on the Montreux Definition. The NARDS group and non-NARDS group were then compared to establish a prediction nomogram for early prediction of NARDS. The predictive accuracy and compliance of the model were evaluated using subject operating characteristic curves, area under the ROC curve, and calibration curves, and the model performance was estimated by self-lifting weight sampling. The Hosmer–Lemeshow test was used to assess the goodness of fit of the model.

**Findings:**

NARDS group consisted of 104, non-NARDS group consisted of 238 newborns in our study. Gestational age, triple concave sign, blood glucose measurement after birth (Glu), Apgar score at the 5th minute (Apgar5), neutrophil count (ANC) and platelet count (PLT) are independent predictors of NARDS in late preterm and term newborns who present with progressive respiratory distress and require varying degrees of respiratory support within the first 24 h of life to minimize work of breathing and restore organismal oxygenation. The area under the ROC curve was 0.829 (95% CI = 0.785–0.873), indicating the model's strong predictive power. In addition, decision curve analysis showed that the model had significantly better net benefits.

**Conclusion:**

In this study, a predictive column-line plot was constructed based on six clinically accessible conventional variables. Early application of this model has a better predictive effect on the early diagnosis of NARDS, thus facilitating more timely and effective interventions.

## Introduction

Neonatal acute respiratory distress syndrome (NARDS) is a severe, life-threatening condition frequently encountered in neonatal intensive care units (NICUs), affecting up to 1.5% of neonates (1, 2). The risk of NARDS increases with decreasing gestational age, making preterm infants the most vulnerable group (3).However, NARDS also frequently occurs in term or near-term infants in clinical practice (4–6). In the neonates populations, pulmonary diseases, including pneumonia and lower respiratory tract infections, remain common risk factors for NARDS (1, 2, 7). Due to the immaturity of the innate immune system in neonates, their ability to defend against pulmonary infections is limited (8, 9), making them more susceptible to infectious pulmonary diseases during the neonatal period. Evidence suggests that pulmonary infections can decrease the biosynthesis of surfactant or impair its secretion, ultimately leading to the formation of hyaline membranes and alveolar collapse, which contributes to the development of NARDS (10–12). Furthermore, inflammation can induce mechanical damage to type II alveolar epithelial cells, thereby further reducing the secretion of pulmonary surfactant (13).

The etiology and pathogenesis of NARDS remain unclear, and there is currently no specific treatment. Management primarily involves supportive care, with intratracheal surfactant administration being the mainstay of treatment until sufficient endogenous surfactant synthesis occurs (4).Therefore, early identification and proactive intervention are crucial for reducing mortality and improving the prognosis of NARDS.

Existing literature has proposed predictive models for NARDS in late preterm and term infants, as well as predictive models for other complications associated with NARDS (14–16). However, the risk factors for pneumonia-related NARDS in newborns born after 34 weeks of gestation remain insufficiently studied. In our study, we integrated key clinically relevant factors, such as gestational age and the triple-concave sign, to develop a predictive model. This model significantly enhances sensitivity and clinical utility for early intervention and improved management strategies in affected neonates when compared to the established PRISM scoring system, which necessitates the inclusion of a broader range of variables.

## Methods

### Study design and population

Infants were admitted to the NICU with difficulty breathing, low oxygen levels, or bluish discoloration of the skin, either occurring right after birth or as a result of various risk factors. They subsequently experienced worsening respiratory distress within the initial 24 h of life. The x-rays revealed air bronchography and reticulonodular shadows throughout the entire lung field, commonly known as a “hair glass” image.

Our hospital's NARDS treatment protocol prioritizes correcting hypoxia, managing the primary disease, and reducing pulmonary artery hypertension through an integrated approach encompassing respiratory support, surfactant replacement, nutritional support, and fluid management. Infants at risk of respiratory acidosis due to asphyxia or hypoxemia receive respiratory support, with HFOV, NIPPV, and NCPAP being our institution's preferred modes. Sedation is provided for infants who are combative with the ventilator.Patients with NARDS typically require more aggressive respiratory support, including mechanical ventilation and PS therapy, which are generally not required for non-NARDS patients, who can meet their oxygen needs via nasal cannula or face mask. For those exhibiting grunting or difficulty breathing with a transcutaneous oxygen saturation below 90% without oxygen supplementation, NCPAP is initiated; surfactant treatment is administered if NCPAP pressure reaches ≥6 cmH2O or FiO2 exceeds 0.30. The combination of HFOV and surfactant can reduce the duration of mechanical ventilation and improve outcomes. Severe NARDS cases may require repeated surfactant dosing. Restricted fluid management is implemented to prevent fluid overload and pulmonary edema, while maintaining hemodynamic stability and tissue perfusion. All patients who were registered were administered ampicillin and a triple cephalosporin to target particular bacterial diseases such as *Group B streptococcus* (GBS) and *Escherichia coli* (E. coli), in accordance with the antibiotic usage guidelines of the NICU at our hospital.

**Figure 1:**
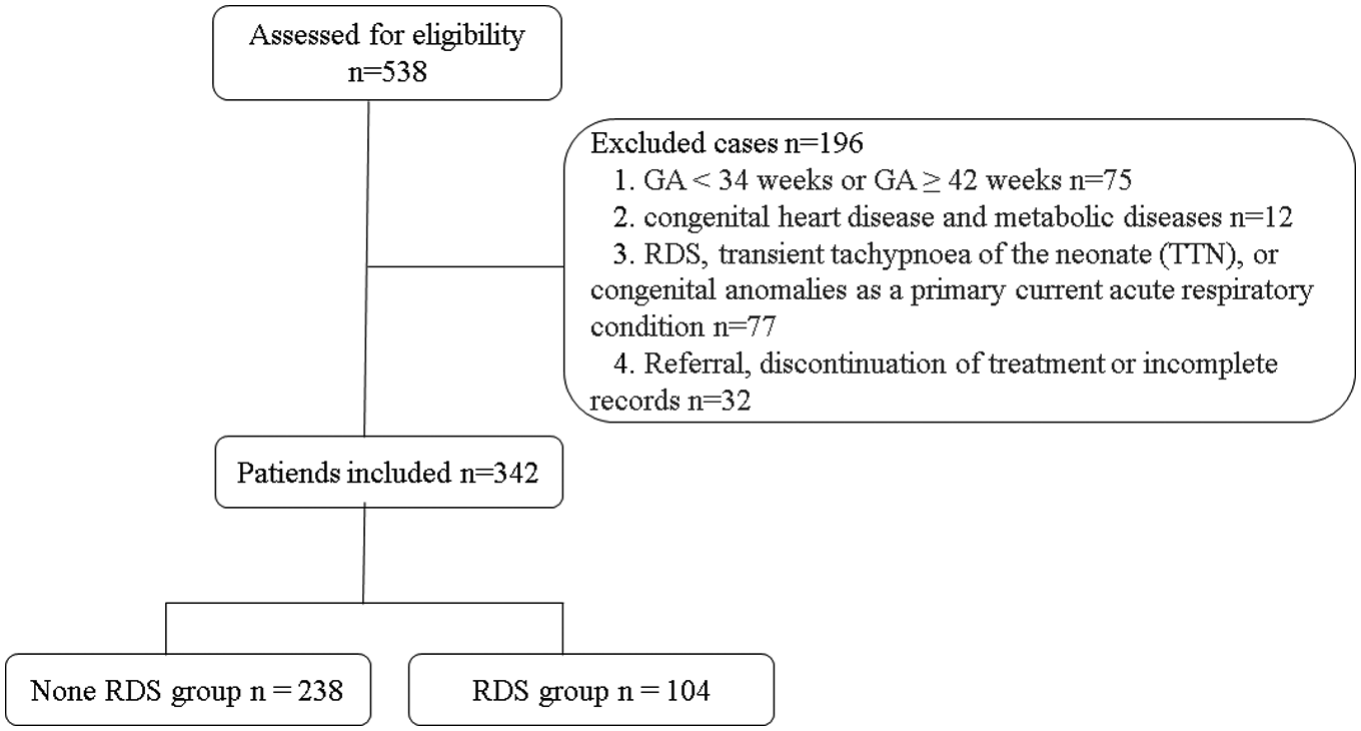
Flow diagram for the present study.

The inclusion criteria were as follows: (a) 34 weeks ≤ GA < 42 weeks. (b) 1,500 g ≤ birth weight ≤ 4,000 g. (c) Progressive respiratory distress necessitating varying levels of oxygen assistance or mechanical ventilation within 24 h of delivery. The exclusion criteria were as follows:(a).GA < 34 weeks or GA ≥ 42 weeks, (b) birth weight <1,500 g (c) congenital malformations (pulmonary adenomatous malformations, diaphragmatic hernia, etc.), RDS and TTN as primary acute respiratory disorders caused by dyspnea, (d) inherited endocrine and metabolic disorders (e) abandonment of treatment by the family or incomplete records. The flow chart is shown in Figure 1.

### Clinical definitions

The diagnostic criteria for NARDS are based on the Montreux definition and require the fulfillment of the following five criteria (2): (1) sudden onset of symptoms within one week after a known or suspected clinical event; (2) ruling out respiratory distress caused by other conditions such as neonatal respiratory distress syndrome (NRDS), transient tachypnea of the newborn (TTN), or congenital abnormalities; (3) lung imaging showing widespread irregularities with reduced clarity, presence of fluid, or areas of opacity that cannot be explained by other factors such as localized fluid accumulation, collapsed lung, NRDS, TTN, or congenital abnormalities; (4) no presence of congenital heart disease that could explain the fluid accumulation in the lungs (this includes cases where the ductus arteriosus is causing overflow of blood into the lungs, unless there is acute pulmonary bleeding); (5)oxygenation deficit is measured as the Oxygenation Index (OI). Mild NARDS is defined as an OI between 4 and 8. Moderate NARDS is defined as an OI between 8 and 16. Severe NARDS is defined as an OI of 16 or higher.

All patients in the NICU are required to undergo arterial blood gas analysis and dynamic monitoring upon admission. Unfortunately, our hospital's electronic medical record system does not routinely document parameters related to neonatal oxygenation, which prevented us from assessing the levels of OI in this study, thereby affecting our analysis of the relationship between the severity of NARDS and the studied parameters. We diagnosed NARDS based on relevant clinical and laboratory findings at admission, strictly adhering to the 2017 Montreal criteria. Furthermore, all subjects included in the study were confirmed by a consensus between two associate chief physicians; any subjects with discrepancies between the two were excluded to ensure the scientific rigor and accuracy of our research.

### Data collection

The clinical data and laboratory findings of newborn pneumonia were obtained from the electronic medical record system in our hospital. (1) Key factors considered for the newborns includeed gestational age, birth weight, sex, 1 min Apgar score, 5 min Apgar score, and the presence of the triple concave sign. In a quiet state of a newborn, especially during inhalation, observe whether there are significant depressions in the three areas of the suprasternal fossa, supraclavicular fossa, and intercostal spaces. Additionally, when assessing the triple concave sign, we combine it with other clinical manifestations, such as rapid breathing and expiratory grunting, to ensure the accuracy of the assessment. (2) The following tests were conducted: routine blood tests, liver and renal function tests, cardiac enzyme tests, and arterial blood gas tests. These tests included measuring neutrophil counts, lymphocyte counts, hemoglobin levels, platelet counts, PCO2 levels, glycemia levels, lactate levels, creatinine levels, albumin levels, aspartate aminotransferase levels, alanine aminotransferase levels, lactate dehydrogenase levels, total bile acids levels, and serum total bilirubin levels. (3) Maternal health was also evaluated, taking into account the mode of delivery, potential contamination of amniotic fluid, maternal fever due to infection within seven days prior to delivery, premature rupture of membranes, signs of fetal distress, gestational diabetes, and any hypertensive disorders experienced during pregnancy. (4) Clinical outcomes, including transfusion therapy, duration of noninvasive and mechanical ventilation, and length of hospitalization. Routine blood samples were obtained at the time of NARDS diagnosis, and the results are typically available within 24 h in accordance with established international biochemical laboratory methods.

### Statistical analysis

In this study, we utilized SPSS statistical software to perform Levene's test and Shapiro–Wilk test to assess the homogeneity of variance and normality of the data, respectively. Initially, we accessed “Analyze” → “Compare Means” → “One-Way ANOVA” from the menu bar, dragged the dependent variable into the dependent list, and placed the grouping variable in the factor box. Within the options settings, we checked “Homogeneity of Variance Tests,” then ran the analysis and interpreted the output results. If the *p*-value of Levene's test is less than 0.05, it indicates that there is unequal variance between groups. Subsequently, the steps for the Shapiro-Wilk test involved selecting “Analyze” → “Descriptive Statistics” → “Explore,” placing the variable to be tested in the dependent list, and in the options, checking “Normality tests with plots.” The *p*-value of the Shapiro-Wilk test was then examined. If it is less than 0.05, it suggests that the data do not conform to a normal distribution. These two tests provide a necessary statistical foundation for subsequent data analysis, ensuring the reliability of the experimental design and results. At the same time, we use VIF values to detect collinearity between the independent variables, ensuring that all VIF < 5 to mitigate the impact of skew data on the analysis results.

To evaluate the stability and generalizability of our model, we employed a bootstrapping procedure for internal validation. Specifically, bootstrapping provides a distribution of model performance through multiple resampling iterations, helping us understand how the model performs across different samples, thereby effectively assessing its robustness and predictive capability. The study employed 1,000 resampling iterations. In each iteration, samples were randomly drawn from the original dataset to generate a bootstrap sample of the same size as the original dataset. A predictive model was trained on each bootstrap sample, and the performance metrics recorded (such as accuracy and sensitivity) were summarized to comprehensively assess the predictive capability of the model.

Data that followed a normal distribution were represented as mean ± standard deviation (X + SD), while skewed data were represented using IQR (interquartile range). Categorical counts were given as the number of instances (%). Distinct samples *T*-tests were conducted to analyze data that followed a normal or approximately normal distribution. Mann–Whitney *U*-tests were employed for comparing groups when the data was skewed. Chi-square tests or Fisher's exact tests were utilized for analyzing categorical variables.

Significant characteristics were incorporated into multivariate logistic regression models to determine independent risk factors for acute respiratory distress syndrome in infants. The independent risk factors identified by multivariate logistic regression analyses were represented as column-line graphical models using R software. The Hosmer-Lemeshow test was employed to evaluate the adequacy of the model's fit. The model's prediction accuracy and conformity were assessed utilizing subject work features such as ROC curves, area under the ROC curve (AUC), consistency index (C-index), and calibration curves. Decision curve analysis (DCA) demonstrated the overall advantage of the model for patients in terms of net benefit.

All statistical procedures were performed using SPSS Statistics 25.0 for Windows (IBM Corp., Armonk, NY) and R software version 4.4.0 for Windows (R Foundation for Statistical Computing, Boston, MA, United States). A level of *P* < 0.05 was considered significant.

## Results

### Comparison of overall data and laboratory parameters between the two groups

This study examined clinical data from 538 instances of neonatal pneumonia observed from January 2021 to December 2023 in newborns who primarily displayed respiratory distress. During the study period, a total of 342 instances of newborn pneumonia were examined. The Montreux Definition was utilized to diagnose NARDS, leading to the classification of 104 cases in the NARDS group and 238 instances in the non-NARDS group. Table 1 compares the demographic and clinical characteristics between NARDS and Non-NARDS groups. Statistically significant differences were observed in newborn weight, Apgar5 min, triple-concave sign, length of hospital stay, CPAP, mechanical ventilation duration, blood transfusion, gestational age, glucose levels, C-reactive protein, neutrophils count, platelet count between the two groups (*P* < 0.05).

**Table 1:** Comparison of overall data and laboratory parameters between the two groups.

Variables	None NARDS group (*n* = 238)	NARDS group (*n* = 104)	*P*
Basic demographics			
Male, *n* (%)	142 (59.7)	62 (60.2)	0.927
Birth weight, grams	2,750 (2,260,3,250)	2,270 (1,955,3,000)	0.001
Apgar1 min	9 (8,9)	8 (8,9)	0.408
Apgar5 min	9 (9,10)	9 (8,9)	0.000
X3 signs, *n* (%)	154 (64.7)	96 (92.3)	0.000
Clinical outcomes			
Hospital stay, days	8 (7,10)	14 (12,17)	0.000
CPAP, days	2.0 (0.0,3.0)	3.0 (2.0,4.0)	0.000
Ventilator, days	0.0 (0.0,0.0)	4.0 (2.5,6.0)	0.000
Blood transfusion, *n* (%)	16 (6.7)	30 (28.8)	0.000
Maternal delivery information			
GA, weeks	36.3 (35.2,38.6)	34.3 (34.0,36.8)	0.000
Caesarean delivery, *n* (%)	161 (67.9)	74 (71.8)	0.473
PROM, *n* (%)	90 (37.8)	34 (32.7)	0.365
Amniotic fluid pollution, *n* (%)	45 (18.9)	14 (13.5)	0.220
Intrauterine distress, *n* (%)	20 (8.4)	14 (13.5)	0.150
HDP, *n* (%)	52 (21.8)	26 (25)	0.523
GDM, *n* (%)	76 (31.9)	31 (29.8)	0.697
Fever, *n* (%)	19 (8)	4 (3.8)	0.160
Primipara, *n* (%)	184 (77.3)	74 (71.2)	0.224
Multiple pregnancies, *n* (%)	25 (10.5)	16 (15.4)	0.201
Laboratory test results			
PO2, mmHg	91 (70,118)	96 (66,123)	0.542
PCO2, mmHg	35 (28,43)	40 (30,47)	0.064
Lac, mmol/L	2.2 (1.5,3.5)	2.3 (1.7,3.3)	0.724
Glu, mmol/L	4.4 (3.8,5.2)	3.5 (3.1,4.7)	0.000
-BE, mmol/L	5.7 (4.0,7.8)	6.8 (4.2,9.0)	0.656
HCO3, mmol/L	19 (18,21)	19 (17,21)	0.210
CRP, mg/L	0.2 (0.2,1.1)	0.2 (0.2,0.5)	0.008
ANC, ×10^9^ /L	7.2 (4.6,11.2)	5.3 (3.6,9.3)	0.005
ALC, ×10^9^ /L	3.5 (2.8,4.5)	3.6 (2.7,5.1)	0.179
HB, g/L	173 (161,186)	167 (155,183)	0.134
PLT, ×10^9^ /L	240 (203,290)	229 (200,249)	0.000
Cr, umol/L	56 (47,66)	57 (47,63)	0.625
ALT, U/L	10 (7,15)	9 (7,13)	0.113
AST, U/L	54 (42,79)	54 (46,77)	0.676
LDH, U/L	517 (437,649)	501 (446,643)	0.577
TBA, umol/L	8 (6,13)	9 (7,13)	0.169
TBIL, umol/L	37(31,46)	37(32,43)	0.381
ALB, g/L	35(33,38)	34(32,37)	0.148

Abbreviations: X3, signs triple-concave sign; CPAP, continuous positive airway pressure; GA, gestational age; PROM, premature rupture of membrane; HDP, hypertensive disorders of pregnancy; GDM, gestational diabetes; Fever, fever within 7 days before delivery; PO2, partial pressure of oxygen; PCO2, partial pressure of carbon dioxide; Lac, lactic acid; Glu, blood glucose; -BE, base excess; HCO3, bicarbonate; CRP, C-reactive protein; ANC, neutrophil count; ALC, lymphocyte count; HB, hemoglobin; PLT, platelet count; Cr, creatinine; AST, aspartate transaminase; ALT, alanine transaminase, LDH, L-lactate dehydrogenase; TBA, total bile acid; TBIL, total bilirubin; TP, total protein; ALB, albumin.

### Selection of the predictive factors by multivariate logistic regression analysis

Based on univariate and multivariate logistic regression analysis, we identified six independent risk factors associated with the occurrence of NARDS (Table 2). These factors include gestational age (*P* = 0.000, OR 0.498, 95% CI 0.376–0.659), triple concave sign (*P* = 0.000, OR 4.451, 95% CI 1.920–10.316), blood glucose (*P* = 0.001, OR 0.682, 95% CI 0.543–0.855), Apgar5 (*P* = 0.005, OR 0.680, 95% CI 0.521–0.888), neutrophils count (*P* = 0.005, OR 1.138, 95% CI 1.040–1.245), and PLT (*P* = 0.035, OR 0.994, 95% CI 0.988–1.000).

**Table 2:** Univariate and multivariable logistic regression analysis for the predictive factors.

Characteristics	Univariate analysis		Multivariate analysis	
	OR (95%CI)	*P*	OR (95%CI)	*P*
GA, weeks	0.616(0.529,0.718)	0.001	0.498(0.376, 0.659)	0.001
X3 signs, *n* (%)	6.545(3.035,14.118)	0.001	4.451(1.920,10.316)	0.001
Apgar5 min	0.613(0.465,0.808)	0.001	0.680(0.521, 0.888)	0.005
Glu, mmol/L	0.614(0.503,0.749)	0.001	0.682(0.543, 0.855)	0.001
Birth weight, grams	0.999(0.999,1.000)	0.001	None	None
ANC, ×10^9^ /L	0.931(0.884,0.980)	0.006	1.138(1.040, 1.245)	0.005
PLT, ×10^9^ /L	0.990(0.985,0.995)	0.001	0.994(0.988,1.000)	0.035
TP, g/L	0.950(0.921,0.979)	0.001	None	None
ALB, g/L	0.947(0.899,0.997)	0.038	None	None

Abbreviations: X3, signs triple-concave sign; GA, gestational age; Glu, blood glucose; ANC, neutrophil count; PLT, platelet count; TP, total protein; ALB, albumin.

### Nomogram development and predictive value of the prediction model in NARDS

**Figure 2:**
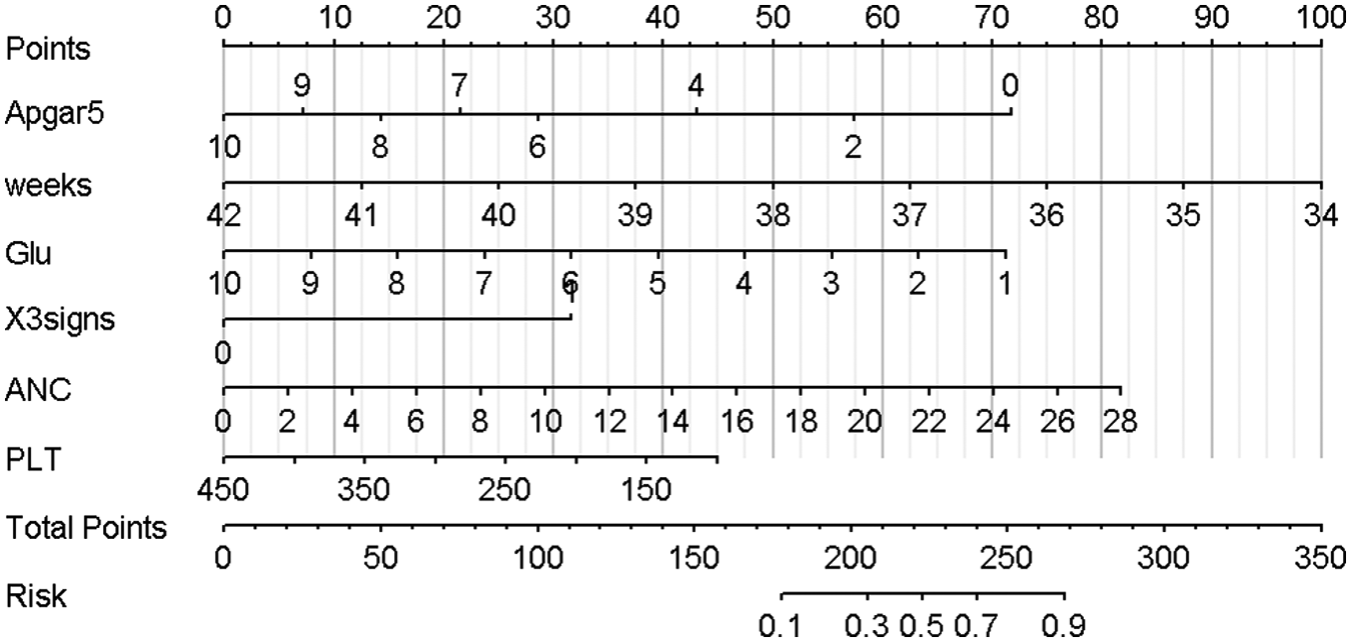
Nomogram for predicting NARDS. Instructions for using the nomogram: **(A)** Draw a line perpendicular from each of the six variables to the top line labeled “Points” to obtain the corresponding number of points; **(B)** add the points obtained from each of the six variables to obtain the total number of points; and **(C)** draw a line descending from the axis labeled “Total points” until it intercepts the Risk. The risk value corresponding to the Risk represents the specific risk at which NARDS will occur.

**Figure 3:**
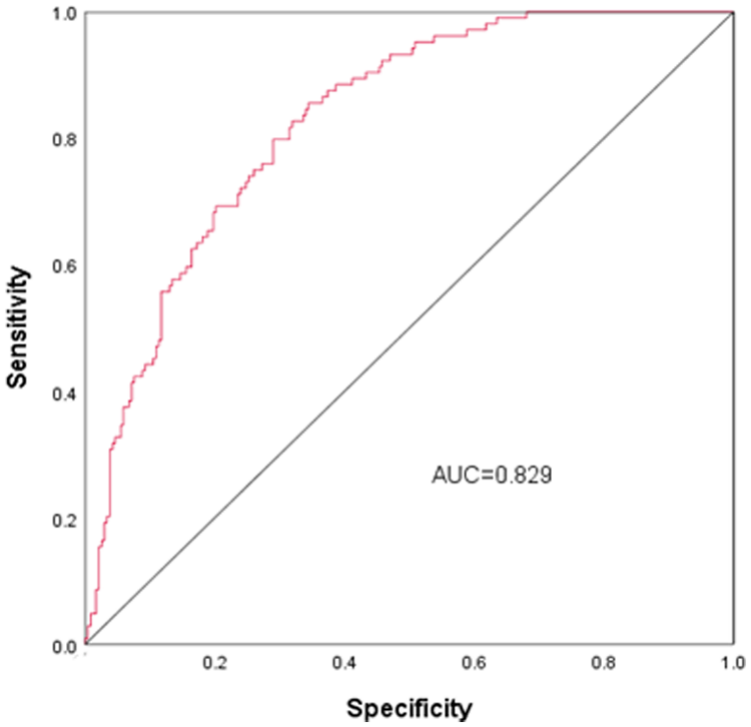
ROC curves for NARDS. The ROC curve is a tool for assessing the performance of a prediction model, where the accuracy, sensitivity, and specificity of the model are calculated by comparing the actual results to the predicted results. The points on the ROC curve indicate the cutoffs for the predicted results, and the closer the area under the ROC curve (AUC) is to 1, the better the predictive performance of the model. The AUC (95% CI) of this nomogram was 0.829 (0.785–0.873).

**Figure 4:**
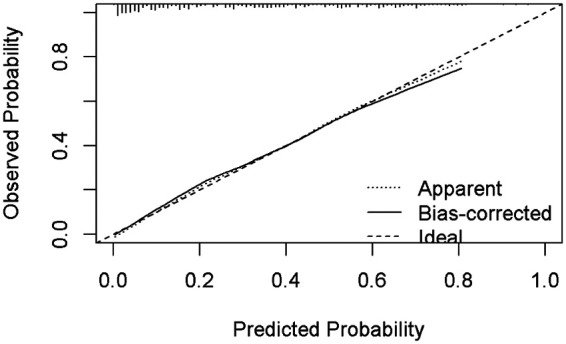
Calibration curve for predicting probability of NARDS model. The nomogram model to predict NARDS is plotted on the x-axis, and the actual NARDS is plotted on the y-axis. The reference line is 45° and indicates perfect calibration. The calibration curve in our model closely approximated a straight line, demonstrating that the prediction model exhibits excellent fit and reliability.

**Figure 5:**
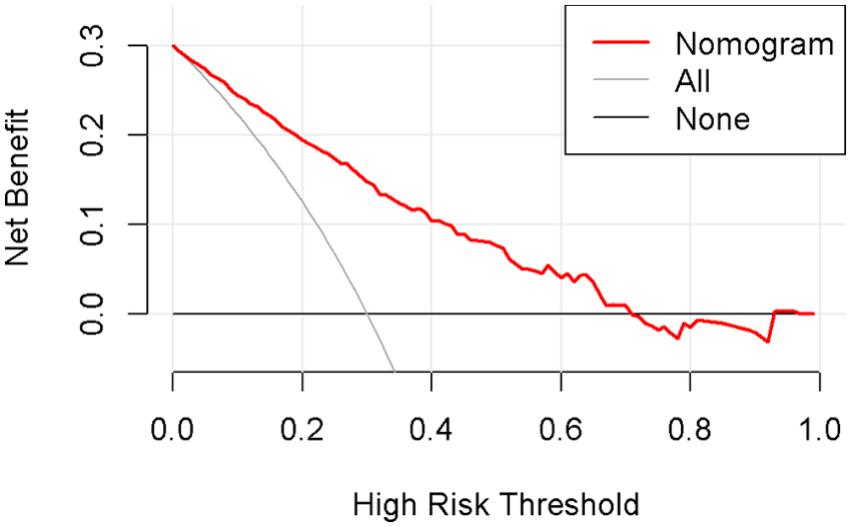
Decision curve analysis in prediction of NARDS model. The *x*-axis represents the threshold probability, while the *y*-axis indicates the corresponding net benefit. The curves in the graph indicate the clinical utility of the predictive model at specific thresholds; a higher curve suggests that the model provides better decision support within the corresponding threshold range.

Using the outcomes of the multivariate logistic regression analysis explained earlier, a conclusive prediction model for NARDS was created and shown as a column-line graph (Figure 2). The cumulative scores of gestational age, triple concave sign, blood glucose, Apgar5, neutrophils count, and PLT in this column-line graph were found to be correlated with a higher likelihood of developing NARDS. Greater cumulative scores imply an increased likelihood of infant NARDS. The area under the ROC curve (AUC) approaches 1, indicating better predictive performance of the model. In this study, our model achieved an AUC of 0.829, with a 95% confidence interval of 0.785–0.873 (Figure 3). This result suggests that our predictive model demonstrates good discriminative ability in predicting NARDS. We further employed the Hosmer–Lemeshow goodness-of-fit test to assess the model's calibration, yielding a result of *p* = 0.858, which indicates a good fit. Additionally, the calibration curve for predicting NARDS closely approximated the ideal curve, providing further support for the model's validity (Figure 4). In clinical decision-making, the DCA curve demonstrated significant net benefits (Figure 5), confirming that our predictive model has substantial practical value in clinical practice.

## Discussion

NARDS is a severe medical illness that significantly endangers the lives of neonates (1, 17, 18).Given the intricate nature of the causes and development of NARDS, coupled with the uncertainty in treatment options (19)—since there is currently no specific remedy—early diagnosis is critical. This study found that gestational age, the presence of the triple concave sign, glucose levels (Glu), Apgar score at 5 min (Apgar5), neutrophil count (ANC), and platelet count (PLT) are significant factors in predicting the development of NARDS in children born between the late preterm and term periods. And our work is the first to develop a predictive model for newborn pneumonia with NARDS after 34 weeks.

Gestational age is a crucial determinant of neonatal lung maturity and overall respiratory function. Infants born prematurely often exhibit underdeveloped lungs, which leads to surfactant deficiency, increased respiratory effort, and a heightened risk of developing Neonatal acute respiratory distress syndrome (NARDS). This underscores the importance of gestational age in assessing respiratory outcomes in neonates. The results of our investigation demonstrated that gestational age was a significant and separate factor in predicting the occurrence of NARDS, and it was incorporated into the predictive model. As the gestational age decreases, the nomogram model score increases, indicating a larger likelihood of NARDS incidence. According to Lin C et al (20), gestational age is a significant factor that affects the likelihood of developing NARDS. The study found that for each additional week of gestation, the risk of NARDS decreased by 60% for newborns who got prenatal steroid medication and by 40% for those who did not receive it. The study conducted by De Luca R et al (21) found that infant mortality and morbidity had a significant correlation with gestational age, with the lowest occurrence observed between 38 and 40 weeks of gestation, regardless of the method of delivery. Our data strongly support the idea that gestational age is a reliable predictor for the development of NARDS in neonates.

NARDS is defined by the presence of pulmonary edema caused by an elevated permeability of the alveolar-capillary barrier, leading to reduced arterial oxygenation (22). The triple-concave sign, characterized by three distinct concavities observed during physical examination, can serve as an important indicator of significant respiratory failure. Neonates exhibiting varying levels of respiratory distress often present with this sign, suggesting a correlation with underlying lung pathology that provides valuable insights into their respiratory condition. A study conducted on animals demonstrated that hypoxia causes a reduction in pulmonary phospholipids and lung surfactant proteins, a decrease in the density of blood vessels between the air sacs, an increase in the permeability of capillaries, and the creation of hyaline membranes in fetal rats (23). These findings support the hypothesis that the triple-concave sign may be a significant predisposing factor for the onset of NARDS. In this study, we performed a multifactorial logistic regression analysis and determined that the triple-concave sign is an independent predictor of NARDS. Its incorporation into our predictive model demonstrates strong predictive power, underscoring its clinical relevance.

In addition to the well-known stress-induced hyperglycemia, hypoglycemia may also reflect a pathological acute stress response (24). Prior research has indicated that mild hypoglycemia in persons who are in good health triggers pro-inflammatory, pro-atherosclerotic thrombosis, and pro-coagulant reactions (25).Tottman AC et al. (26) colleagues found that the hypoglycemia group had a higher prevalence of serious newborn abnormalities compared to the normoglycemic and hyperglycemic groups. Furthermore, the presence of low blood sugar levels was linked to the seriousness of the illness and higher death rates in individuals with sepsis (27). Considering the negative impact of hypoglycemia on clinical results, we incorporated blood glucose into the prediction model. Our study revealed that the baseline glucose level was significantly lower in the group of patients with NARDS compared to those without NARDS. In the final multivariable logistic regression analysis, we found that blood glucose (OR <1) is a protective factor for NARDS, indicating that lower blood glucose levels are associated with a higher risk of NARDS. The following pathophysiological mechanisms can explain the occurrence of hypoglycemia: Firstly, critical illness in infants can lead to significant changes in energy intake and nutritional requirements (28). Premature infants, being in a state of relative catabolism and insulin resistance, exhibit more pronounced manifestations of hypoglycemia (29, 30). Secondly, newborn infections and the release of pro-inflammatory cytokines lead to increased peripheral glucose utilization and inhibition of gluconeogenesis, resulting in hypoglycemia (31–33).

The Apgar score is a globally accepted and standardized metric for assessing the condition of newborns immediately after birth, particularly their respiratory effort, heart rate, and overall responsiveness. A low Apgar score reflects significant perinatal stress and acute hypoxia, which is often associated with severe neonatal asphyxia. This condition can impair oxygenation and ventilation, contributing to the pathophysiology of NARDS. Research has shown that a low Apgar score at 5 min is linked not only to infant mortality but also to poor prognoses in severe neurological and non-neurological diseases, as well as organ malfunction (34–36). Furthermore, a study conducted by Ernest E et al. (37) found a strong correlation between low Apgar scores at 5 min and the subsequent development of respiratory diseases in infants and adolescents. Additionally, low Apgar scores have been strongly associated with an increased likelihood of cerebral palsy and epilepsy in children under 16 years of age (38). Our findings suggest that lower Apgar scores at 5 min correlate with an increased risk of NARDS. This association is indicative of a higher likelihood of newborn hypoxemia, which can lead to energy depletion, oxidative stress, inflammation, and ultimately cell death (39). During episodes of hypoxia, blood redistribution occurs, resulting in reduced perfusion to the lungs, which may cause varying degrees of lung damage, including respiratory distress, pulmonary hemorrhage, elevated pulmonary pressures, and potentially respiratory failure (40). These insights underscore the importance of early identification and management strategies for affected neonates to mitigate the risks associated with low Apgar scores.

One important characteristic of NARDS is the buildup of neutrophils in the lungs. The activation and recruitment of neutrophils are believed to have a crucial impact on the advancement of NARDS (22, 41). Neutrophils are the initial immune cells that are called upon to gather at areas of damage or inflammation. They possess strong antimicrobial properties, such as oxidants, proteases, cationic peptides, and neutrophil extracellular traps (42, 43). Shen L (16)demonstrated that integrating neutrophils into a joint prediction model resulted in a more accurate prediction of early diagnosis of NARDS. Our research indicates that absolute neutrophil count (ANC) can serve as a reliable marker for early detection of NARDS. The odds ratios associated with ANC in both univariate and multivariate logistic regression analyses appear contradictory, likely due to potential confounders that were not controlled for in the univariate analyses, such as age, sex, and disease severity. In contrast, the multivariate logistic regression analysis revealed a positive correlation between ANC and the risk of NARDS, suggesting a complex immunomodulatory mechanism underlying the body's response to infection. Given the intricate relationship between ANC and infection, future studies should aim to integrate additional potential biomarkers to further investigate the interactions among various factors and enhance the predictive power regarding NARDS outcomes.

Platelets are small, anucleated cell fragments of around 2–4 μm in diameter. They are produced by megakaryocytes and are discharged into the bloodstream (44).They have a primary role in triggering a response to vascular damage and thrombosis, and they also play a crucial role as mediators in the blood (45, 46).Platelets engage in interactions with various cell types in the blood and endothelium, generating a diverse array of powerful cytokines and chemokines that play a role in immunological responses and thrombosis (47). Consequently, this leads to a reduction in the number of platelets in the peripheral blood. The results of our investigation indicate a decrease in platelet count during the initial phases of neonatal pneumonia when paired with NARDS. These findings align with the research conducted by Shen L et al (16).Research has shown that the breakdown of platelet mitochondrial membranes, elevated levels of cytoplasmic calcium, and increased phosphatidylserine presentation are associated with a decrease in the number of platelets in the peripheral blood (48, 49).

### Limitations

It is important to acknowledge that this study was a retrospective analysis conducted at a single center, with a relatively small sample size, which may affect the broader applicability of the model. Relying solely on internal validation to assess the model may limit its generalizability. Additionally, we did not evaluate the potential impact of the severity of NARDS on the experimental outcomes. To further ensure the reliability and validity of the findings, future prospective multicenter studies are needed. Nonetheless, this research utilized easily accessible clinical indicators and employed a robust and scientific data processing methodology, ensuring the accuracy of the model. This lays the groundwork for future multicenter validation studies and the inclusion of additional biomarkers, while also holding significant implications for guiding clinical improvements in neonatal prognosis.

## Conclusions

To summarize, this study discovered that gestational age, triple concave sign, Glu, Apgar5, ANC, and PLT were important indicators of NARDS in infants born at a later stage of pregnancy or at full term. Using these indicators, we developed a predictive model for NARDS and then verified its accuracy to ensure it was a good fit. The utilization of this nomogram model might serve as a dependable instrument for physicians to promptly identify and acknowledge NARDS, hence playing a crucial role in mitigating the elevated fatality rate linked to NARDS.

## Data Availability

All the data used and analyzed during the current study are available from the corresponding author upon reasonable request.
